# Ethical and Methodological Considerations of Twitter Data for Public Health Research: Systematic Review

**DOI:** 10.2196/40380

**Published:** 2022-11-29

**Authors:** Courtney Takats, Amy Kwan, Rachel Wormer, Dari Goldman, Heidi E Jones, Diana Romero

**Affiliations:** 1 City University of New York School of Public Health New York City, NY United States

**Keywords:** systematic review, Twitter, social media, public health ethics, public health, ethics, ethical considerations, public health research, research topics, Twitter data, ethical framework, research ethics

## Abstract

**Background:**

Much research is being carried out using publicly available Twitter data in the field of public health, but the types of research questions that these data are being used to answer and the extent to which these projects require ethical oversight are not clear.

**Objective:**

This review describes the current state of public health research using Twitter data in terms of methods and research questions, geographic focus, and ethical considerations including obtaining informed consent from Twitter handlers.

**Methods:**

We implemented a systematic review, following PRISMA (Preferred Reporting Items for Systematic Reviews and Meta-Analyses) guidelines, of articles published between January 2006 and October 31, 2019, using Twitter data in secondary analyses for public health research, which were found using standardized search criteria on SocINDEX, PsycINFO, and PubMed. Studies were excluded when using Twitter for primary data collection, such as for study recruitment or as part of a dissemination intervention.

**Results:**

We identified 367 articles that met eligibility criteria. Infectious disease (n=80, 22%) and substance use (n=66, 18%) were the most common topics for these studies, and sentiment mining (n=227, 62%), surveillance (n=224, 61%), and thematic exploration (n=217, 59%) were the most common methodologies employed. Approximately one-third of articles had a global or worldwide geographic focus; another one-third focused on the United States. The majority (n=222, 60%) of articles used a native Twitter application programming interface, and a significant amount of the remainder (n=102, 28%) used a third-party application programming interface. Only one-third (n=119, 32%) of studies sought ethical approval from an institutional review board, while 17% of them (n=62) included identifying information on Twitter users or tweets and 36% of them (n=131) attempted to anonymize identifiers. Most studies (n=272, 79%) included a discussion on the validity of the measures and reliability of coding (70% for interreliability of human coding and 70% for computer algorithm checks), but less attention was paid to the sampling frame, and what underlying population the sample represented.

**Conclusions:**

Twitter data may be useful in public health research, given its access to publicly available information. However, studies should exercise greater caution in considering the data sources, accession method, and external validity of the sampling frame. Further, an ethical framework is necessary to help guide future research in this area, especially when individual, identifiable Twitter users and tweets are shared and discussed.

**Trial Registration:**

PROSPERO CRD42020148170; https://www.crd.york.ac.uk/prospero/display_record.php?RecordID=148170

## Introduction

Since its launch in 2006, Twitter has become one of the most popular social media sites as a platform that allows users to post and interact with short messages known as tweets. According to a 2019 survey by Pew Research Center [1], 1 in 5 (23%) adults in the United States report using Twitter. While Twitter users are not representative of the general population (users tend to be younger, more educated, and located in urban or suburban areas) [2], the volume of publicly available tweets allows for research to be conducted on large data sets, eschewing a common perceived limitation of small samples.

Public health researchers have identified “big data” from Twitter as a new wellspring from which research can be conducted [3]. However, the utility of these data depends on the appropriateness of the research questions and the methodological approaches used in sampling and analyzing the data. Previous systematic reviews have explored how Twitter data have been used. A systematic review by Sinnenberg et al [4] of 137 articles using Twitter in health research between 2010 and 2015 found that the main research questions explored with Twitter data involved content analysis, surveillance, engagement, recruitment, intervention, and network analysis. Similarly, a scoping review from 2020 [5] found 92 articles that fell within 6 domains: surveillance, event detection, pharmacovigilance, forecasting, disease tracking, and geographic identification. Additional systematic reviews of social media, beyond Twitter alone, have examined specific domains, for instance, exploring how these data, including Twitter, are being used for public health surveillance [6-8] or pharmacovigilance [9-11].

While social media provides new opportunities for data sources in research, some unique obstacles are also present. For instance, the presence of spam and noisy data can make it difficult for researchers to identify a legitimate signal for the research topic in question [12]. To navigate this issue, researchers sometimes opt to employ traditional manual coding of content; however, this can be a nonideal solution given the size of the data sets and the time and effort required for these analyses [13]. Other teams have used natural language processing (NLP) or machine learning approaches, which present their own problems; one study [14] found that among the algorithms built to classify emotions, the highest performing model had an accuracy of 65%. The landscape of social media necessitates understanding of the mechanisms and limitations of the platforms, as well as adaptations to the requirements of this landscape.

In addition to the research questions and methodological approaches used with Twitter data, the extent to which social media data are in general considered public, and what this means for ethical research oversight are unclear. There is substantial literature discussing the ethics of using social media data for public health research, but clear ethical guidelines have not been established [15-24].

The need for these guidelines is increasingly pressing, as leveraging social media for public health research raises questions about privacy and anonymity; properly deidentifying user data requires the researchers to understand an “increasingly networked, pervasive, and ultimately searchable dataverse” [18]. Information shared on social media can often be intensely personal; hence, anonymity would be even more important for research involving sensitive data such as health conditions and disease [23]. This is particularly relevant for the field of public health, since the data collected and analyzed for public health research will often fall into these more sensitive categories.

Beyond the questions of user anonymity, when conducting research on more sensitive health information, traditional research protocols center the importance of informed consent among participants. However, there are currently no established guidelines for the expectation of consent when leveraging publicly available social media data. Some theorists in the realm of internet research ethics have proposed an assessment model that determines the need for consent based on possibility of pain or discomfort. They further suggest that this assessment should consider the vulnerability of the population being studied and the sensitivity of the topics [22].

In the systematic review by Sinnenberg et al [4], approximately one-third of the 137 articles included therein mentioned ethical board approval. Given that Twitter usage has changed dramatically in recent years [25], this systematic review is an updated examination of both ethical considerations and research questions or methodologies across all domains of public health research using Twitter.

We sought to investigate the methodological and ethical aspects of using Twitter data for public health research from 2006, when Twitter was launched, to 2019 [26]. Specifically, we describe the measures being used in Twitter research, the extent to which they are validated and reliable, and the extent to which ethical oversight is included in studies using publicly available tweets.

## Methods

### Design

This review followed the PRISMA (Preferred Reporting Items for Systematic Reviews and Meta-Analyses) guidelines [27,28] and was registered with PROSPERO (CRD42020148170).

### Eligibility Criteria

The database search was limited to peer-reviewed public health studies originally written in English, which were published between January 2006 and October 31, 2019, and used social media data to explore a public health research question. The social media platforms included in the search were Twitter and Sina Weibo (China’s version of Twitter), Facebook, Instagram, YouTube, Tumblr, or Reddit.

Studies were excluded if they were systematic or literature reviews, marketing or sales research, only investigated organizational-level tweets, investigated tweets from conferences in disciplines other than public health, or included primary data collection asking participants about their social media use. We excluded articles that focused on organizations disseminating information to the public (evaluation of social media dissemination and analysis of organizational- or institutional-level social media data) or testing interventions that used social media as a method (intervention study using social media), as our research question was not related to interventions using social media platforms as a tool but rather explored how existing social media data are being used in secondary analyses in public health research.

Given the volume of studies identified, separate analyses were conducted on Facebook and YouTube; thus, this systematic review focuses solely on Twitter. Studies that included Twitter and other social media platforms were included, but only Twitter findings were extracted.

### Information Sources

We searched PubMed, SocINDEX, and PsycINFO for articles about social media and public health after consulting with our institutional librarian on the best approaches to the search.

### Search

The search strategy consisted of the Boolean search term: ((“Social media” OR twitter OR tweet* OR facebook OR instagram OR youtube OR tumblr OR reddit OR “web 2.0” OR “public comments” OR hashtag*) AND (“public health” OR “health research” OR “community health” OR “population health”)).

### Study Selection

**Figure 1:**
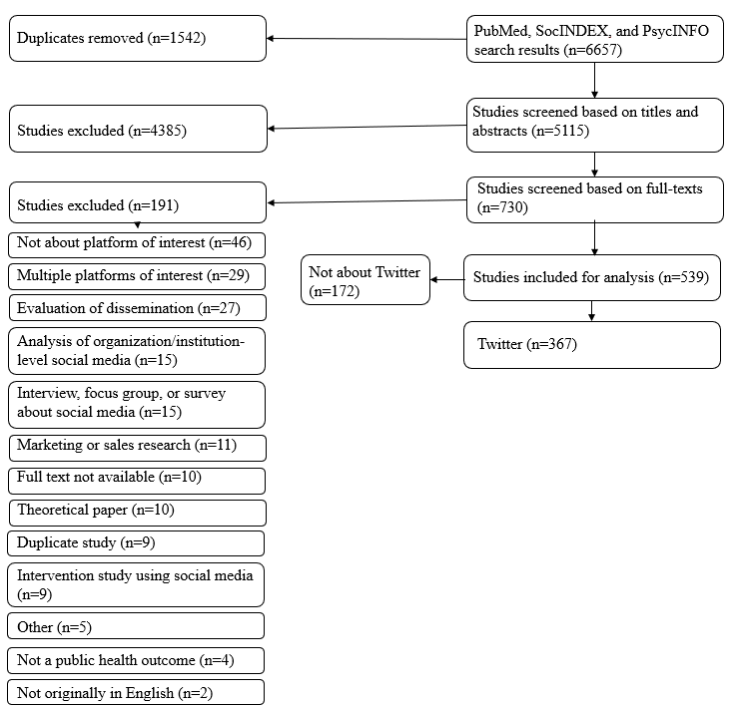
PRISMA (Preferred Reporting Items for Systematic Reviews and Meta-Analyses) flowchart for systematic review of methodological approaches and ethical considerations for public health research using Twitter data, 2006-2019.

Three authors reviewed abstracts for eligibility in a 2-step process, with each abstract reviewed by 2 authors independently. A first screen was performed on the basis of the title and abstract; if deemed ineligible, the study was excluded from further screening. Disagreements were resolved through discussion and consensus. Full texts of the remaining articles were retrieved for the second screen and reasons for exclusion were coded and ranked by the priority of exclusion criteria for cases in which more than one exclusion criterion was applied (Figure 1). Disagreements about inclusion and exclusion criteria were resolved through discussion and consensus.

### Data Collection Process

Data were extracted using a standardized data extraction spreadsheet, which was developed a priori and refined during the data extraction process. This refinement resulted in the removal of data elements; new data elements were not added. To establish consistency in extractions, 2 reviewers independently extracted data from the same 5 articles and compared the results. This process continued during weekly meetings, in which papers of varying complexity were discussed until consensus was reached. No studies were excluded on the basis of their quality.

### Data Items

The data items in this review categorized information about the study within 4 domains: (1) study characteristics: public health topic, year, and country of publication; (2) study design and results: sample size, Twitter data extraction method, operationalization (ie, which data points were collected from social media posts and how researchers quantified these data), methodologic and analytic approaches, primary results, and descriptions of linking or account data; (3) ethical considerations: ethical approval, discussion of informed consent, and general discussion of ethical issues; and (4) risk of bias or methodological checks: quality assessment, validity, reliability, and accuracy checks implemented. We defined methodological approach as the overall objective of a research project coupled with the operationalization of methods to fulfill this objective.

Quality assessment metrics were adapted from existing quality assessment tools used for systematic reviews [29-31]. The specific quality assessment metrics were the following: whether the stated research question matches the data-defined research question, the presence of a clearly defined objective or hypothesis, validity of measures, reliability of measures, validation of computer algorithms, whether the data analysis is sufficiently grounded, whether findings logically flow from the analysis and address the research questions, and the presence of a clear description of limitations. A study was considered to have addressed validity if the measures used were based on validated measures, previous studies, or existing frameworks. A study addressed reliability if manual coding efforts incorporated checks or assessed intercoder reliability, descriptions of reliability were not expected for studies that only used machine learning. Accuracy checks were described if manual checks were performed by researchers or validation of computer algorithms used for studies using machine learning algorithms and NLP.

### Summary Measures

The summary measures related to methods and study design include the following: the frequency of studies by topic, geographic focus, year of publication, analytic approach, sampling approach, and overall methodological approach or objective of the study (ie, surveillance, content exploration, sentiment mining, network science, and model development and testing). The summary measures related to ethical considerations include the frequency of studies that sought institutional review board (IRB) review or approval, included informed consent from Twitter handlers, discussed ethical considerations within the paper, and reported identifying results (ie, verbatim tweets). For quality assessment, we present information on the validity and reliability of measures used; a full summary of quality assessments is provided in Multimedia Appendix 1.

## Results

Our search resulted in 6657 unique studies for review, of which 730 required full-text review (Figure 1). We identified 539 studies across all social media platforms; 367 used Twitter data forming the analytic sample for this review (Multimedia Appendix 2 for the full list of included articles with all data extraction fields; for readability of text, references are only included when details of specific articles are provided as contextual examples).

### Study Characteristics

#### Public Health Research Topics

The most common public health topics among the articles reviewed were communicable diseases (eg, influenza, Ebola, and Zika; n=80, 22%), substance use (n=66, 18%), health promotion (n=63, 17%), chronic disease (eg, cancer; n=48, 13%), and environmental health (n=48, 13%; Multimedia Appendix 1).

#### Year of Publication

**Figure 2:**
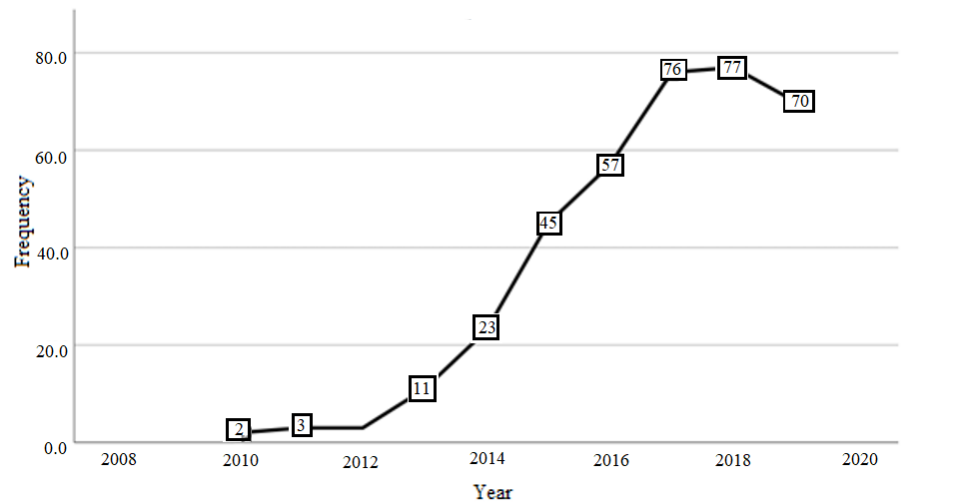
Number of articles published by year for systematic review of methodological approaches and ethical considerations for public health research using Twitter data, 2006-2019.

The year of publication for the articles in this review ranged from 2010 to 2019. A sharp increase in the number of Twitter articles was observed from 2012 to 2017 (Figure 2). Two preprint articles on October 31, 2019, were included in the count for 2019 [32,33].

#### Geographic Focus

Most studies analyzed tweets originating from the United States (n=158, 43%) or worldwide (n=134, 36%); only 75 (20%) of them focused on non-US regions or countries. Of the articles that had a global geographic focus, 23 (17%) of them collected geotags and reported on geospatial metrics within the body of the article. Despite having a worldwide focus, these 23 articles demonstrated a bias toward the United States, western Europe (namely the United Kingdom), Canada, and Australia; the majority of the data collected in these studies were posts originating in these countries, with a distinct minority representing other regions or countries.

### Study Design and Results

#### Sample Size and Unit of Analysis

Of the 367 articles reviewed here, 355 (97%) used individual tweets as the unit of analysis and 11 (3%) used Twitter accounts (or “handles”) as the unit of analysis. One article (0.3%) used keywords as the unit of analysis, as the study sought to identify keywords that would help researchers detect influenza epidemics via Twitter [34].

There was a wide range of sample sizes. For studies with tweets as the unit of analysis (n=353), the number of analyzed tweets ranged from 82 [35] to 2.77 billion [36] (median=74,000), with 90 papers having a sample size larger than 1 million. Similarly, for studies using Twitter handles as the unit of analysis (n=11), the sample size ranged from 18 [37] to 217,623 [32].

#### Methods for Accessing Data

To pull data from Twitter, most studies used application programming interfaces (APIs) that were developed by Twitter (eg, Gardenhose and Firehose) and could be integrated into statistical software packages. Third-party APIs (eg, Twitonomy and Radian6) were also used frequently, either through contracting with a commercial vendor, purchasing tweets that match specified criteria, or using software developed by an entity outside of Twitter. Most studies either mentioned that they used an API without indicating the specific type (37%) or did not mention their method of tweet accession (13%; Table 1). Of papers that identified the API used, purposive and random sampling were equally employed. However, only 22 (7%) articles explicitly mentioned whether the API used was purposive or random in its sampling technique; when the API was named (eg, decahose, search API, and Gardenhose) but the sampling type was not noted in the article, we looked up the sampling technique in use by the API.

We also found that the description of the sampling method was often not described. For instance, some Twitter APIs are purposive in nature (eg, Twitter Search API) and some are random (Twitter Firehose API) or systematic (some REST APIs). Many studies did not specify what type of sampling was used to extract tweets from Twitter or did not fully explain retrieval limitations (eg, how it might affect the sample population if only a certain number of tweets could be retrieved daily through an API).

**Table 1:** Frequency of studies by access method and data source from a systematic review of methodological approaches and ethical considerations for public health research using Twitter data, 2006-2019.

Method or source for Twitter data		Frequency (N=367), n (%)
**Access method**		
	Unspecified application programming interface (API)	136 (37)
	Purposive sampling^a^	88 (24)
	Random sampling^a^	84 (23)
	Existing database	10 (3)
	Unspecified method of accession	49 (13)
**Data source**		
	Native Twitter API/functionality	222 (60)
	Third-party API	102 (28)
	Unknown	34 (9)
	In-house program	9 (3)

^a^Accession methods and sampling type are differentiated as random or purposive in accordance with reports from the articles’ authors or Twitter.

#### Methodological Approach

As seen in Table 2, the most common methodological approaches were as follows: thematic exploration (eg, describing the themes of conversations about e-cigarettes on Twitter) [38], sentiment mining (eg, assessing if tweets about vaccines are positive, negative, or neutral) [39], and surveillance (eg, tracking the patterns of information spread about an Ebola outbreak) [40]. Less common methodological approaches were tool evaluation (eg, using Twitter data to predict population health indices) [41] and network science (eg, examining health information flows) [42]. Different methodological approaches tended to be pursued for different topics. For example, most infectious disease research was in the domain of surveillance, whereas research about mental health and experiences with the health care system was more conducive to thematic exploration and sentiment mining.

Across the 3 most common study methodological approaches (thematic exploration, sentiment mining, and surveillance), approximately one-third of the papers (36%) used machine learning (Table 2). Machine learning here is defined as an application of algorithms and statistical modeling to reveal patterns and relationships in data without explicit instruction (eg, to identify the patterns of dissemination related to Zika virus–related information on Twitter) [43]. This can be contrasted to NLP, which necessitates explicit instruction; often, NLP is used to identify and classify words or phrases from a predefined list in large data sets (eg, to identify the most common key topics used by Twitter users regarding the opioid epidemic) [44]. Of the articles reviewed, NLP was more prevalent in sentiment mining than in other types of methodological approaches.

**Table 2:** Frequency of studies by methodological approach and analytical technique from a systematic review of methodological approaches and ethical considerations for public health research using Twitter data, 2006-2019.

Methodological approach and analytical technique^a^		Frequency (N=367), n (%)
**Sentiment mining**		227 (62)
	Natural language processing	145 (64)
	Machine learning	66 (29)
	Spatial analysis	12 (5)
	Descriptive analyses or frequencies	4 (2)
**Surveillance**		224 (61)
	Natural language processing	104 (46)
	Machine learning	85 (38)
	Spatial analysis	17 (8)
	Descriptive analyses or frequencies	18 (8)
**Thematic exploration**		217 (59)
	Natural language processing	114 (52)
	Machine learning	81 (37)
	Spatial analysis	13 (6)
	Descriptive analyses or frequencies	9 (4)
	Tool evaluation	61 (16)
	Network science	36 (10)

^a^Multiple responses were allowed.

### Ethical Considerations

#### Presence of Identifying Information

Just under half (n=174, 47%) of the articles reviewed did not contain any identifying information of Twitter accounts or tweets, 36% (n=131) of them contained anonymized account information or paraphrased tweets, and 17% (n=62) of them contained direct quotes of tweets or identifiable information such as Twitter handles or account names (Table 3). Of the 62 articles that included verbatim tweets or identifying information about the user, one-third (n=21, 34%) of them included a discussion of ethics in the paper (eg, Berry et al [45]).

Less than half of the articles (n=173, 47%) indicated that they did not use any of the metadata (eg, username, demographics, and geolocation) associated with the tweet (Multimedia Appendix 1). Approximately one-third of the articles (n=110, 30%) used geographic information associated with the tweet, and a much smaller number of articles (n=15, 4%) included photos associated with the account or health information (such as illness disclosure or mentions of medications taken). Of the articles analyzing tweets from either the United States or another specific region or country (n=233), 37% (n=86) of them used geotags of Twitter accounts to identify the location of the tweets; of the articles that did not specify a geographic region (n=134), 17% (n=23) of them used geotagging.

Though research on infectious disease and health promotion were most likely to include user metadata in their data analyses, linked health information was most often used in papers about infectious disease and mental health, often in the form of medical self-disclosures.

#### IRB Approval and Informed Consent

Just under one-third of the articles reviewed (n=119; 32%) explicitly stated that those studies sought and received IRB review or approval (Table 3). The majority (n=226, 61%) of them did not mention IRB approval, although many of these articles included statements about the nature of Twitter posts being publicly available. Only a small subset (n=23, 6%) of studies explicitly stated that IRB approval was not necessary.

Among those that sought IRB approval (n=119), over half (n=68, 57%) of them were granted exemptions; just under half (n=49, 41%) of them did not specify the type of approval received. Two studies [46,47] received full IRB approval. One of them [46] retrospectively examined existing public data about health beliefs regarding the human papillomavirus and was approved with a waiver of consent owing to its retrospective design. The other study [47] had 2 parts: study 1 consisted of a survey of self-reported stress following a school lockdown, and study 2 consisted of data mining of community-level rumor generation during the lockdown on Twitter. The survey necessitated informed consent as it involved human participants; hence, the full scope of the study (parts 1 and 2) had to undergo IRB review. None of the studies using only Twitter data sought informed consent, even when including identifying information from Twitter handlers or tweets. Over two-thirds of the articles (n=258, 70%) did not include a discussion of ethics or privacy concerns.

Additionally, 53 (49%) articles discussed the anonymization of data used in their study either by omitting usernames and Twitter handles [48] or by providing only paraphrased tweets to prevent exact-match searching [49]. Only 5 studies included specific and extensive discussions around the ethical implications of social media research and went beyond disclaimer statements about the publicly available nature of tweets. One study [50] described consulting guidelines for internet research from various organizations and researchers, while another [51] included a long “ethical considerations” section that described needing to “weigh threats to safety and privacy against benefits gained by using novel approaches to study suicide,” and acknowledged vulnerable populations and risks of stigma and discrimination. Another study [52] raised the challenge of social media research given the lack of relevant ethical frameworks.

**Table 3:** Frequency of studies by ethics-related factors from a systematic review of methodological approaches and ethical considerations for public health research using Twitter data, 2006-2019.

Ethics-related factors				Frequency (N=367), n (%)
**Level of identification**				
	No identifying information		174 (47)	
	Anonymized data and paraphrased tweets		131 (36)	
	Identifiable information and direct quotes		62 (17)	
**Institutional review board (IRB) approval obtained**				
	Yes		119 (32)	
	No		23 (6)	
	Not mentioned/unclear		225 (61)	
**Among those with IRB approval (n=119)**				
	Exempt		68 (57)	
	Nonexempt		2 (2)	
	Not specified (eg, “approved”)		49 (41)	
**Informed consent of Twitter handler attempted**				
	Yes		0 (0)	
	No		119 (100)	
**Any discussion of ethical considerations, including disclaimers**				
	**Yes^a^**		109 (30)	
		Discussion of anonymization process	53 (49)	
		Extensive discussion^b^	5 (5)	
		Other discussion, including disclaimers	54 (49)	
	No		258 (70)	

^a^Note that 3 articles included both an extensive discussion of ethics as well as details regarding their anonymization process.

^b^The denominator for the articles that discussed ethics is 109.

### Risk of Bias in Individual Studies

We found that 270 (74%) articles included a clear description of the validity of measures; 21 (6%) articles were purely exploratory in nature and collected only counts of tweets, so we deemed them exempt from an assessment of validity of measures; 76 (21%) articles did not include efforts at establishing measurement validity. Further, of the 264 articles involving human coding, 184 (70%) included a description of intercoder reliability and quality assurance checks, while 80 (30%) did not. Similarly, 235 articles involved computer algorithms or automated coding, of which 165 (70%) explicitly described accuracy checks or validation of the algorithms, while 70 (39%) did not.

In addition to concerns about validity and reliability of measures, one of the main sources of bias was the sampling frame. The self-selection of Twitter users was discussed in most of the studies, with 85% (n=314) of them describing this as a potential limitation.

## Discussion

### Principal Findings

#### Summary Measures

We saw evidence of a steep increase in publications using Twitter data after 2012, which may be due to Twitter releasing its native standard (version 1.1) API in 2012, which made mining of its data much more accessible to the general public without the need for complex coding capabilities [53]. The prevalence of research using “big data” from Twitter is increasing and will likely continue to do so in the coming years [50].

Infectious disease was the most common topic of the research papers, which may indicate a burgeoning interest in using social media to detect disease outbreaks. It is likely that a review of studies using Twitter data that picks up from where this study left off (ie, after October 31, 2019) would support this finding given the onset of the COVID-19 pandemic in late 2019.

There are some major considerations that this review highlights for the future of public health research using Twitter data. Most of the research focused on Twitter users in the United States; this includes the articles with a global focus that demonstrated a bias toward the anglophone world. Three articles appeared to genuinely have a representative global scope; interestingly, two of these were about the Zika virus. This indicates the data scraped from Twitter tends to be heavily focused on the United States and English-speaking settings.

Another major consideration is that of the accession method used to build a data set. Most of the studies examined in this review used APIs or variations thereof; only 10 studies used alternative accession methods. Those 10 studies used data either extracted from Twitter for previous studies or hosted in pre-existing databases. Of the remaining studies that used an API, only 22 studies explained whether the API used was purposive or random in nature. This is of interest because the sampling technique of APIs has been called into question in previous papers [54,55]. In particular, the Twitter Streaming API is considered to produce less representative samples and should be approached with caution; this API is susceptible to intentional or accidental bias based on inclusion and exclusion criteria selected for a particular study [56]. Owing to the “black box” nature (ie, lack of documentation of the sampling approach) of native Twitter APIs, it cannot be determined that data retrieved using Twitter APIs are truly random [57,58].

In addition to the aforementioned obstacles, there are questions about the accuracy of algorithms using machine learning and NLP. A little less than half of the papers reviewed for this systematic review involved surveillance and prediction, and approximately one-sixth of them evaluated new tools or frameworks in the realm of Twitter data. Machine learning was commonly used for these methodological approaches. However, a previous evaluation of the efficacy of using various machine learning algorithms to automatically identify emotions expressed on Twitter found that the highest performing algorithm achieved an accuracy rate of 65% [14]. Another recent article found that machine learning was not effective in making meaningful predictions about users’ mental health from language use on social media; further, Twitter metadata and language use was not specific to any one mental health condition [59].

This raises concerns about the overall use of social media data for research, as data science in general and public health research in particular use data to make insights; these data “then get acted upon and the decisions impact people’s lives” [20]. Hence, conscientious planning is advised when using publicly available social media data for the purpose of public health research.

#### Discussion of Ethics

Given that slightly over one-third of studies anonymized Tweets or Twitter users, many researchers seem to think that there are ethical considerations when using these data, even if they are publicly available. Nevertheless, the majority of projects did not seek IRB review or approval. This contradiction suggests an implicit understanding that while there are no international or place-specific ethical guidelines around research using social media data, there is something unique about the nature of this research that distinguishes it from truly public data.

International ethical standards for biomedical and public health research already exist, and these standards often continue to influence the national guidelines that develop within a given country [60-62]. Given the global scope of social media, it may be most prudent for guidelines to be established on an international scale and then adapted to place-specific committees and ethics boards. However, this is complicated by the ever-evolving landscape of social media use and data agreements. The field of research ethics has yet to fully address the introduction of new media as sources of data; even before a comprehensive international framework is introduced, it may be advisable for institutions and regions to enact their own interim frameworks to mitigate possible harm and preserve user privacy and anonymity to the extent possible.

### Limitations

This systematic review has a number of limitations. Owing to the iterative nature of data extraction for a large number of articles included, it is possible that there were differences in how data were coded as we refined our process. However, we attempted to minimize this concern through weekly research team meetings during the extraction process. Another limitation is that because we only examined articles originally published in English, we may be underestimating the number of articles that were conducting research in a specific geographic area other than the United States. The influence of this underestimation should be minimal; however, as most leading journals for health research are published in English [63]. One final limitation is that the literature review spanned from 2010 to 2019, so we are not capturing changes since then, which may have taken place in the approach to ethics or methodology in research using social media data since then. This is an evolving field of research; hence, we anticipate that standards and norms may have also evolved.

### Comparison With Prior Work

Similar to Sinnenberg et al’s [4] review, this study examined whether ethics board approvals were sought when using social media data for public health research, finding equivalent proportions of articles that obtained IRB approval. Our study further explored whether there were other types of ethical considerations (eg, ethical discussion) present in the body of the articles. We also assessed the presence and use of identifiable information such as personal health information, verbatim Tweets, and user account metadata. In both this review and in that of Sinnenberg et al [4], many articles noted that the public nature of tweets allows researchers to observe the content. This presents a clear need for an ethical guideline framework for researchers using Twitter, especially when including identifying information.

### Conclusions

Twitter data appear to be an increasingly important source of data in public health research. However, attention needs to be paid to sampling constraints, ethical considerations involved in using these data, and the specific methodologies to be used to ensure the rigorous conduct of this research.

## Appendix

Multimedia Appendix 1 Supplementary tables.

Multimedia Appendix 2 Full data extraction sheet.

## Abbreviations

API: application programming interface
IRB: institutional review board
NLP: natural language processing
PRISMA: Preferred Reporting Items for Systematic Reviews and Meta-Analyses
