# Supplementary material for: Ethical and Methodological Considerations of Twitter Data for Public Health Research: Systematic Review
Source: J Med Internet Res. 2022 Nov 29;24(11):e40380. doi: 10.2196/40380 (PMC9748795; doi:10.2196/40380)
Supplement: Multimedia Appendix 1 [file jmir_v24i11e40380_app1.docx]

Count of articles satisfying each quality assessment metric

| Quality Metric | Satisfy Criterion  n (%) | Valid total N |
| --- | --- | --- |
| Stated research question matched data-defined research question | 361 (98.4) | 367 |
| Clearly defined objective/hypothesis | 353 (96.2) | 367 |
| Validity of Measures | 272 (78.8) | 345^a^ |
| Reliability of Measures | 186 (71.3) | 261^a^ |
| Validation of computer algorithms | 167 (71.4) | 234^a^ |
| Data analysis sufficiently grounded | 308 (83.9) | 367 |
| Findings flow from analysis and address research question | 321 (87.5) | 367 |
| Clear description of limitations | 314 (85.6) | 367 |

^a^ N is less than 367 for validity, reliability, and validation checks because not all articles employed relevant methods (i.e., articles without any manual coding were not assessed for reliability)

Frequency of studies by public health topic from a systematic review of methodological approaches and ethical considerations for public health research using Twitter data, 2006-2019.

| Public Health Topic | Frequency (N=367)  n (%) |
| --- | --- |
|  |  |
| Communicable disease | 80 (21.8) |
| Substance use | 66 (18.0) |
| Health promotion | 63 (17.2) |
| Chronic disease | 48 (13.1) |
| Environmental health | 48 (13.1) |
| Mental health | 43 (11.7) |
| Maternal, sexual, and reproductive health | 27 (7.4) |
| Vaccines | 24 (6.5) |
| Healthcare system experience | 16 (4.4) |
| Adverse drug reactions | 12 (3.3) |
| Social determinants of health | 10 (2.7) |
| Healthy community design | 10 (2.7) |
| Health equity | 7 (1.9) |
| Global health | 1 (0.3) |

^a^ Note: These topics are not mutually exclusive, so one paper can cover multiple topics; as such, the total count of Table 1 will be higher than the number of articles reviewed.

Count of articles by linking data within article

| Data Item (N=367) | n (%) |
| --- | --- |
|  |  |
| None | 173 (47.1) |
| Username | 25(6.8) |
| Demographics | 37 (10.1) |
| Network | 6 (1.6) |
| Geolocation | 92(25.1) |
| Username and Geolocation | 18 (4.9) |
| Photos | 2 (0.5) |
| Named influencers | 1 (0.3) |
| Personal Health Information | 13 (3.5) |
